# Per and Polyfluoroalkyl Substances in Tap Water Following an Accidental Release of Fire-Fighting Foam into the Drinking Water System in McKeesport, Pennsylvania, USA

**DOI:** 10.1021/acsestwater.5c01358

**Published:** 2026-02-16

**Authors:** Shan Niu, Ruiwen Chen, Aaron Winchell, Carla Ng

**Affiliations:** † Advanced Interdisciplinary Institute of Environment and Ecology, Guangdong Provincial Key Laboratory of Wastewater Information Analysis and Early Warning, School of Technology for Sustainability, Beijing Normal University, Zhuhai 519087, China; ‡ Department of Civil and Environmental Engineering, 6614 University of Pittsburgh, Pittsburgh, Pennsylvania 15261, United States; § Department of Environmental and Occupational Health, University of Pittsburgh, Pittsburgh, Pennsylvania 15261, United States; ⊥ Department of Chemistry, University of Pittsburgh, Pittsburgh, Pennsylvania 15261, United States

**Keywords:** chemical release, PFAS, community contamination, human exposure

## Abstract

On July 16th, 2021, a fire at an auto body shop in McKeesport, Pennsylvania, caused firefighting foam containing per and polyfluoroalkyl substances (PFAS) to enter the drinking water system. Although the water authority flushed the distribution system, community concerns persisted about PFAS residues in plumbing and contamination risk at consumers’ taps. To address this, we collected tap water from kitchen, bathroom, and laundry sinks in homes during November 2021- May 2023. Point-of-use filters (POUFs) were provided, and filters were collected to evaluate their efficacy. Our results showed that five months after the incident, perfluorooctanesulfonate (PFOS) levels in 5 out of 15 homes exceeded the USEPA’s drinking water threshold of 4 ppt. 6:2 fluorotelomer sulfonate was frequently detected in the tap water. Kitchen taps had the highest PFAS levels, followed by bathroom and laundry taps. GenX was detected in aerator extracts from 5 homes but determining its source requires further investigation. POUFs effectively removed specific PFAS, and hydrant flushing by the local water authority was successful in decreasing levels across the water system. However, lack of clear communication following the event left the community distrustful of their water quality, and community members reported feeling disconnected from the decision-making process.

## Graphical abstract

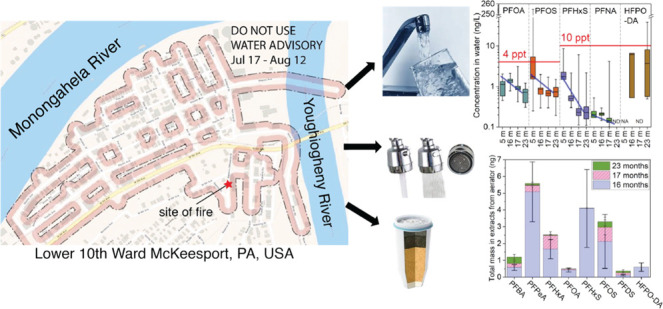

## Introduction

Aqueous film-forming foam (AFFF) is a specialized Class B firefighting agent used for fires involving flammable or combustible liquids. These foams have long contained complex mixtures of per- and polyfluoroalkyl substances (PFAS) as active ingredients.^1^ Due to health concerns associated with PFAS exposure,^2^ the use of PFAS-containing AFFF has been restricted to specific sites, including military sites, airports, and oil and gas facilities. The AFFF used in firefighting and previously in training activities continues to result in drinking water contamination near airports and military sites.^3–5^ Although AFFF is intended for use in such specialized settings, municipal firefighters are occasionally donated surplus foams, which may lead to unintended PFAS contamination when used to fight structure fires. Such an event occurred during a fire at an auto body shop in McKeesport, an environmental justice community^6^ in southwestern Pennsylvania, USA.

On July 16th, 2021, first responders to the auto body shop fire informed the local water authority of a malfunction that caused firefighting foam containing PFAS to be inadvertently drawn through a fire hydrant into the drinking water system. The following day, the Municipal Authority of Westmoreland County (MAWC) issued a “Do Not Use” water advisory affecting 256 residents in the lower 10th Ward of McKeesport (see affected areas in the Supporting Information, Figures S1 and S2). During this time, the MAWC initiated system flushing while awaiting PFAS test results. Independent laboratory tests requested by MAWC detected elevated PFAS levels (as high as 650 ng/L) initially in hydrants and subsequently further into the water system. The highest concentration of total PFAS was as high as 416 ng/L at consumer taps. (https://www.mawc.org/pfas; test report in Figure S3).

In addition, multiple draw samples collected by MAWC and analyzed by an independent laboratory from various fire hydrants and homes, with the last sampling on August 10th, 2021, showed high initial PFAS levels at first flush, followed by substantially lower concentrations after 5 min of flow (Figure S3). This suggests that PFAS could potentially adhere to the plumbing and hydrants, posing a risk of continued contamination. However, no systematic sampling was conducted from residential taps. Previous work on premise plumbing flushing following PFAS contamination suggests that, while flushing reduces PFAS in water effectively, some contamination from adhered PFAS can persist in parts of the distribution system or premise plumbing leading to increasing concentrations detected after periods of stagnation.^7^

PFAS are highly persistent in the environment, and some can accumulate in the human body. PFAS exposure has been linked to a range of adverse health effects, such as elevated cholesterol levels,^8^ immune suppression,^9^ liver damage,^10^ kidney cancer,^11^ and testicular cancer.^12^ Moreover, certain health impacts can occur even at very low PFAS exposure levels. In response, the US Environmental Protection Agency (EPA) has established new regulations for PFAS, setting safe drinking water levels for these contaminants near zero.^13,14^ PFAS are a diverse group of synthetic chemicals with at least one fully fluorinated carbon atom, as defined by the Organization for Economic Co-operation and Development (OECD).^15^ Given their unique chemical properties, PFAS have been widely used in industrial manufacturing and consumer products, such as electroplating, semiconductor manufacturing, nonstick cookware, food packaging, and AFFF, which is one of the key applications for environmental release. The types of PFAS in AFFF vary depending on the year of production and manufacture. For example, AFFF produced by 3 M Company contained perfluorinated carboxylates (PFCAs) from the 1960s to the early 1970s,^16,17^ and perfluorinated sulfonates (PFSAs) from the 1970s until 2001,^18,19^ when 3 M ceased production of AFFF. In addition, fluorotelomers were included in AFFF formulations from 1988 to 2001.^19^ Perfluorooctanesulfonate (PFOS), a fully fluorinated eight-carbon substance belonging to the PFSA subgroup, and perfluorooctanoic acid (PFOA), a fully fluorinated eight-carbon substance belonging to the PFCA subgroup, have been widely detected in groundwater, soils, and fish near AFFF source zones.^1,20,21^ Other PFAS, such as fluorotelomer sulfonates (FTS), perfluorooctane sulfonamidoethanols (FOSEs), and perfluorooctane sulfonamides (FOSAs), have also been detected in groundwater at AFFF-impacted sites.^5^ While PFAS research has produced numerous studies on environmental contamination, there is still limited understanding of PFAS fate within premise plumbing or within distribution pipes in drinking water systems following accidental AFFF releases.

To help addressing community concerns regarding drinking water safety in McKeesport, we conducted a relatively comprehensive sampling in a subset of affected homes between November 2021 and May 2023 (5–23 months after the incident). Specifically, we collected cold tap water from the kitchen, bathroom, and laundry sinks, with two samples collected at each tap: the first draw and after running the water for 5 min. The kitchen tap’s aerator was also removed and soaked in methanol following the first draw to investigate whether PFAS-containing material (e.g., solids released from scale within pipes) was collecting at the tap over time. We also deployed point-of-use filters (POUFs) and collected the used filters at replacement time from homes involved in this study to understand the efficacy of interventions on reducing PFAS exposures over time. A total of 40 PFAS were quantified in all the samples to understand their presence, profiles, and distribution across the sampled taps. This research provides insight into the evolution of community exposure following the incident, evaluates the efficacy of common interventions including point of use filters and distribution system flushing, and helps address public concerns regarding potential PFAS contamination in water due to the incident.

## Materials and Methods

### Household Recruitment

Households were recruited to the study in collaboration with a local nonprofit with community ties, Women for a Healthy Environment, and with the help of two community members who served as project advisory board members and helped develop mailers and flyers that were distributed throughout the community. Unfortunately, there is no official information on the exact number of homes impacted. However, the region affected by the “Do Not Use” water restriction is provided in Figure S1 of the Supporting Information, and a Facebook post by MAWC (Figure S2) states that the number of customers in the lower 10th Ward at the time of the incident was 256. All the participants in this study were from this impacted area. In addition, the direction of the water flows through the distribution system remains protected information that is not publicly available, so gauging the “travel distance” from the hydrant at the incident site was not possible. A total of 32 homes participated in this study, with most homes participating in one of four sampling events (December 21, 2021, November 10, 2022, December 8, 2022, and May 2, 2023), based on residents’ availabilities and interest in continued monitoring. The first through fourth sampling events included 15, 9, 8, and 5 homes, respectively. The number of samples included in each sampling round are documented in the results section. Three homes participated in two separate sampling rounds. In total, 135 tap water samples, 17 aerators, and 19 filter cartridges were collected.

### Chemicals and Materials

Native PFAS standards including PFCAs, PFSAs, perfluorooctane sulfonamides, perfluorooctane sulfonamidoacetic acids, perfluorooctane sulfonamido ethanols, per- and polyfluoroether carboxylic acids, per- and polyfluoroether sulfonic acids, fluorotelomer carboxylic acids, and FTSs were purchased from Wellington Laboratories (Guelph, ON, Canada). All isotopically labeled standards were also purchased from Wellington Laboratories. A full list of native and isotopically labeled PFAS can be found in Tables S1 and S2. Solvents, including methanol (HPLC grade) and ammonium hydroxide (Optima), were obtained from Fisher Scientific (Waltham, MA, USA). 1 L bottles made of high-density polyethylene (HDPE) were purchased from Fisher Scientific (Waltham, MA, USA). Zerowater brand POUFs and replacement filters were obtained from the manufacturer and from local retail outlets.

### Sampling and Storage

Before use, the HDPE bottles were confirmed to have no PFAS contamination and were precleaned by rinsing three times with water and methanol, followed by air-drying. To investigate potential overnight accumulation of PFAS in household plumbing, tap water samples were collected as early as 6–7 am. Initially, approximately 500 mL of tap water was collected from the kitchen. Subsequently, the aerator was removed, immersed in a 50 mL polypropene (PP) tube containing methanol (ensuring the methanol covered the aerator surface). Following this, separate samples of approximately 500 mL tap water were collected from the bathroom and the basement/laundry taps. All three taps were then flushed for 5 min, and a second 500 mL tap water sample was collected immediately after the flushing period at each tap. Ideally, a total of six bottles of tap water samples were collected from each household, except where any taps were unavailable (e.g., if there was no laundry sink). Additionally, if applicable, filters from pitcher filters were collected (all were ones we deployed), wrapped with aluminum foil, and stored in a Ziploc bag. New filter replacements or POUFs for pitchers were provided to participants. Due to variability in usage and uncertainty regarding filter use duration, an estimation of filtered water volume could not be obtained. Therefore, the collected filter samples were used for PFAS pattern comparison only, rather than for quantitative concentration comparisons. Upon collection, water samples, aerator extracts, and filters were transported on ice to the laboratory and promptly stored at −20 °C for subsequent analysis. Participants completed a questionnaire detailing their demographics, water usage, concerns about water quality, and health status after the incident. All participants provided written informed consent, and the research protocol received approval from the University of Pittsburgh Institutional Review Board.

### PFAS Measurement

A total of 40 PFAS (Table S1) was quantified in the water, aerator, and filter samples. The water sample preparation followed the guidelines of EPA Draft Method 1633.^22^ Approximately 500 mL of water samples were extracted and cleaned up using Weak Anion Exchange (WAX) cartridges (Waters, MA. USA). Prior to extraction, isotopically labeled PFAS were added to the sample tubes as surrogates. For detailed information on the chemicals, including the isotopically labeled PFAS, and sample preparation procedures, please refer to Section S1 and Tables S1 and S2 in the Supporting Information. The aerator extracts were concentrated to 100 μL and transferred into LC vials for instrumental analysis. The POUF cartridge was disassembled using precleaned scissors to remove the top mesh. The activated carbon, located at the top, was first collected and thoroughly mixed. Then the same extraction was performed on the resin layer beneath it. Both the activated carbon and resin were prepared according to USEPA Draft Method 1633 for solid samples. Briefly, about 5 g of activated carbon or resin samples were placed in 15 mL polypropylene centrifuge tubes, spiked with 11 isotopically labeled PFAS, and extracted using 0.3% NH4OH/methanol three times with ultrasonication. The extracts were then combined and concentrated before the cleanup process using WAX cartridges. Detailed description of the cleanup procedure is provided in Section S1 in the Supporting Information.

Quantification of PFAS was conducted according to USEPA Draft 2 Method 1633 ^22^ using an ultrahigh performance liquid chromatograph coupled with a triple quadrupole mass spectrometer (UHPLC-MS/MS; Vanquish Flex and TSQ Quantis, Thermo Scientific). Separation was performed on a C18 column (1.7 μm, 50 × 2.1 mm; Waters Acquity UPLC). Mobile phases A and B consisted of 20 mM ammonium acetate and methanol, respectively. Mass spectrometry used the multiple reaction monitoring (MRM) mode. Electrospray ionization (ESI) in negative ionization mode was employed, with settings at 2.5 kV for spray voltage, 325 °C for the ion transfer tube temperature, and 300 °C for the vaporizer temperature.

The limits of detection (LODs) for PFAS were established as the lowest concentrations meeting a signal-to-noise ratio of 3:1 on the instrument. LODs ranged from 0.1 to 2.5 ng/mL (Table S1). Sample analysis was organized into batches of approximately 10 samples each. Within each batch, one field blank and one laboratory blank were included. Trace amounts of PFBA and PFBS were detected in several field and lab blank samples, with concentrations ranging from N.D. to 0.4 ng/sample, which were lower than the method detection limits reported in USEPA Method 1633 Revision A. The reported PFAS concentrations were blank-corrected using the average concentrations of PFBA and PFBS in the blanks. The recoveries of PFAS surrogates ranged from 45 to 127%, comparable to the recoveries reported in USEPA Method 1633 Revision A. The isotopic dilution method was used for quantifying PFAS, yielding recovery-corrected results. Further details regarding instrumental analysis are provided in Section S1 of the Supporting Information.

### Statistical Analyses

Data below the detection limits were replaced by 1/2 of the LOD when calculating the sum of PFAS. A *t*-test was conducted with Microsoft Excel (Version 16.77.1) to compare PFAS concentrations across groups, with a *p*-value of less than 0.05 considered statistically significant.

## Results and Discussion

### PFAS concentrations in Tap Water

Concentrations of targeted PFAS in tap water are summarized in Table S3. During the initial sampling events, conducted five months after the incident and prior to receiving project funding, only kitchen tap water samples were collected, and the aerators were not removed. Focusing first on PFAS for which maximum contaminant limits have been established (Figure 1), this initial sampling revealed PFOS levels in 5 out of 15 homes exceeded the USEPA drinking water threshold for PFOS (4 ppt) set in 2024. In contrast, the concentrations of PFOA, PFHxS, and PFNA were all below their respective USEPA thresholds (4, 10, and 10 ppt);^23^ PFHxS concentrations were highest among these three compounds.

**Figure 1:**
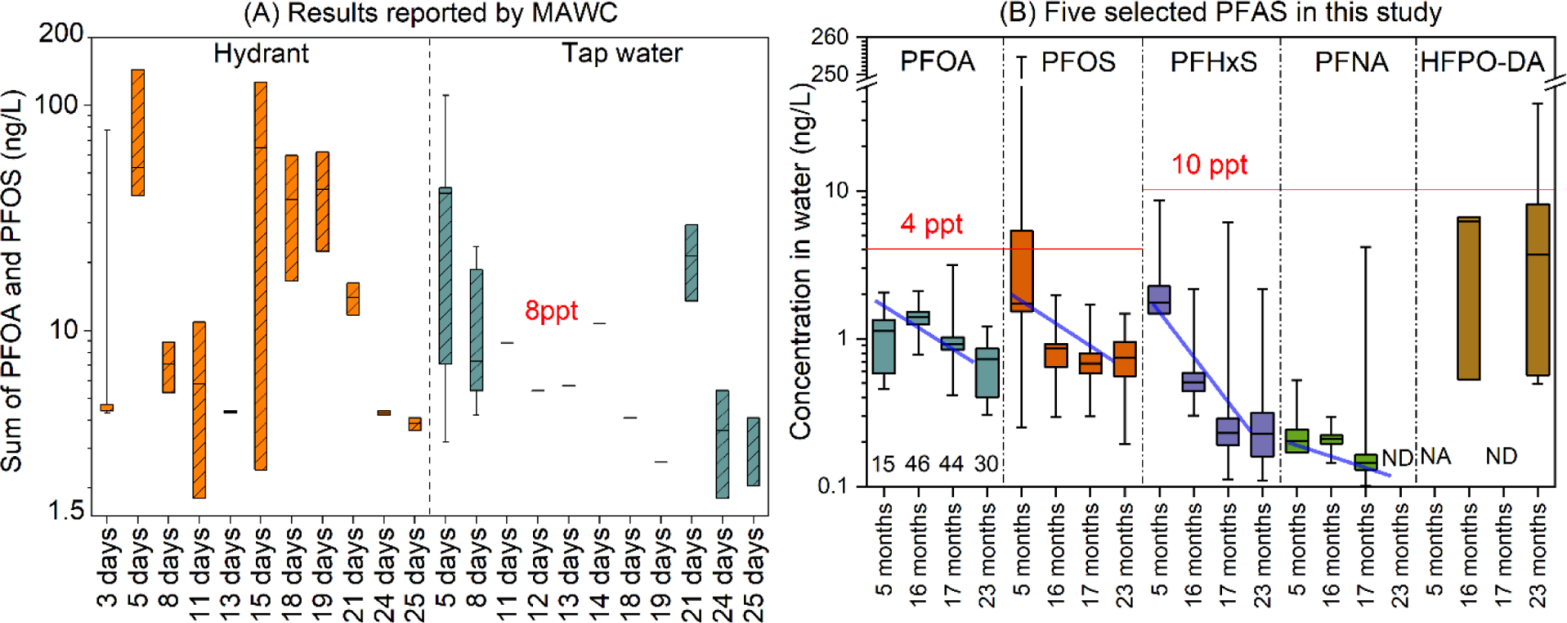
(A) Sum of PFOA and PFOS levels (ng/L) reported by MAWC within 25 days postincident. (B) Concentrations of individual PFAS with established regulatory limits detected in this study (ng/L). The values above the red lines are thresholds for the compounds set by the USEPA in 2024. In Figure 1B, the numbers above *x*-axis labels for PFOA indicate the number of tap water samples collected in each sampling event. ND: not detected; NA: not included in the measurement. Boxes indicate the first and third quartiles, middle lines in the boxes represent median concentrations, and whiskers indicate the maximum and minimum concentrations.

The initial levels of the sum of PFOA and PFOS detected in our samples were consistent with earlier sampling conducted by MAWC (Figure 1A), which were reported as sum PFAS based on Method 533/537 (https://www.mawc.org/pfas). Samples taken by MAWC at hydrants, which are external to the homes and were reported for up to 25 days following the incident, clearly indicate that the distribution system is the source of PFAS to the homes, given that values are equal to or higher than values reported by MAWC at the tap over that same time period. Levels of sum PFOA and PFOS reported in that data set dominated total PFAS in some but not all samples; exact sample composition is unknown. In previous studies, PFOS and PFHxS have been identified as the dominant PFAAs in water near AFFF-impacted areas.^1,21,24,25^

Given the concern regarding the extremely high PFOS levels detected in three homes (254, 153, and 16.8 ng/L respectively) during our first sampling visit, follow-up tap water samples were collected from these homes a few days after the initial measurements (still within the initial “5 months” sampling window, Figure 1B). In this follow-up sampling, PFOS concentrations had decreased to below 4 ppt in all three homes. The reason for the initially high PFOS concentrations in certain houses, followed by substantial decreases, could be that PFOS adhered to the plumbing within the drinking water distribution system, was released sporadically into households, and was subsequently flushed out during water usage. Similar phenomena have been seen in controlled studies after introducing PFAS into a model premise plumbing system.^26^ Although MAWC systematically flushed the water distribution system after the incident, high PFAS concentrations persisted in some hydrants, and samples generally showed high variability (Figure 1A). Consequently, some hydrants near the contamination point were replaced to eliminate them as a continuing source. The three houses with high PFOS concentrations in our study were not those closest to the fire accident site; however, the direction of flow within the drinking water distribution system is unclear and not publicly available due to security concerns. Unfortunately, we did not have access to sampling points outside the homes to confirm the continuing source of PFAS detected at taps (that is, whether some contamination persisted in the distribution system or only in the homes themselves).

Subsequent rounds of sampling between 16 and 23 months following the incident included multiple sampling locations in the home (kitchen, bathroom, and laundry taps as described in the Methods section), and showed consistently lower results for PFOA, PFOS, PFHxS and PFNA compared with time points closer to the time of the fire, with one exception. In the last round of sampling, conducted in May 2023, 23 months after the incident, one sample had an HFPO–DA concentration exceeding the USEPA drinking water threshold of 10 ppt. Notably, HFPO–DA was not included among the targeted PFAS during the initial sampling event because it is not a chemical associated with AFFF.

Among all the targeted PFAS compounds, PFHxA, PFOA, PFOS, and PFHxS were detected in more than 90% of the tap water samples. PFPeA, PFHpA, PFNA, and PFBS were detected in over 70% of the samples, while other compounds were detected in fewer than 35% of the samples. With the exception of three extremely high PFOS levels, the concentrations of the sum of targeted PFCAs, the sum of PFSAs, and 6:2 FTS were generally within a similar range, with medians of PFCAs slightly higher than those of PFSAs (Figure 2). 6:2 FTS was the only fluorotelomer sulfonate (FTSA) detected in all samples (i.e., 4:2 FTS and 8:2 FTS were not detected). In addition, 6:2 FTS is frequently detected in groundwater from areas impacted by AFFF.^21^ In addition, 6:2 FTS can degrade into PFBA, PFPeA, and PFHxA through oxidation,^1^ which may help explain the slightly higher concentrations of PFCAs compared to PFSAs observed in the tap water in McKeesport. In addition, the median concentrations of the sum of PFCAs decreased over time following the incident, a trend that was also observed for the sum of PFSAs. Interestingly, the median concentration of 6:2 FTS showed a slight increase over time. However, none of these decreases or increases were statistically significant. Most of the sums of the concentrations of PFASAs were 10 times lower than those of PFCAs, PFSAs, and 6:2 FTS. The concentration ranges of other PFAS were comparable to those of PFCAs, PFSAs, and 6:2 FTS. However, the detection frequencies of PFASAs and other PFAS were significantly lower than that of PFCAs, PFSAs, and 6:2 FTS.

**Figure 2:**
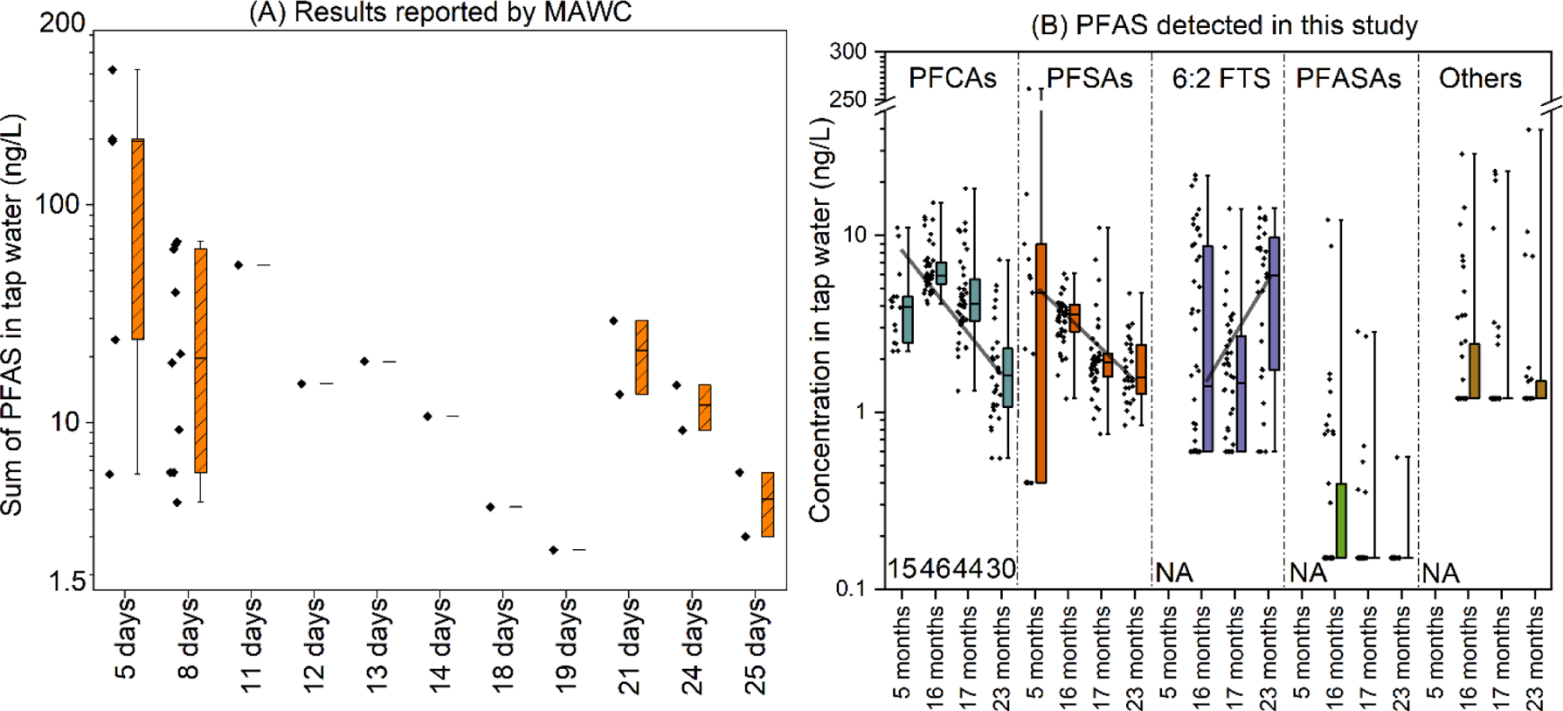
(A) Sum concentrations of PFAS (number of compounds unknown) in tap water reported by MAWC within 25 days postincident (https://www.mawc.org/pfas). (B) Sum of PFAS concentrations (ng/L) within specific PFAS subclasses in tap water detected in this study 5–23 months after the incident. Numbers above PFCA *x*-axis labels indicate the number of samples (not households) in each event. NA means the analysis at that time point did not include that PFAS group. Boxes span first and third quartiles, the middle line the median concentrations, and the whiskers the maximum and minimum concentrations. Black diamonds beside boxes represent all the data.

Overall, decreasing PFAS levels over time indicate that flushing was largely effective in clearing the contaminated water from the drinking water system, though some PFAS remained detectable up to two years following the incident. There was high variability across homes, and the decreasing trend did not hold for 6:2 FTS. These observations suggest that PFAS may indeed persist, to some extent, within pipes as a consequence of adhering to pipe surfaces, as previous work has suggested.^7^ Moreover, recent modeling efforts focused on better understanding premise plumbing flushing following large-scale contamination events^27^ highlight that contaminants may persist in areas such as residential water heaters, and thus a systematic approach that takes into account the specifics of the indoor plumbing design—and employs multiple rounds of flushing—is needed to ensure drinking water safety.

Higher levels of PFAS were observed in water samples collected from kitchen taps compared to those from bathroom and laundry taps in each sampling event (Figure 3), although the differences were not statistically significant. It appears that PFAS did not accumulate within the plumbing inside the houses studied, based on the relatively low concentrations of PFAS found and possibly due to the frequent use of water in the homes alongside the water utility flushing of the system. Another possibility is that PFAS might accumulate in plumbing infrastructure during the first few days following the incident and be flushed out as water usage increases. The profiles of PFAS varied across the three taps: in kitchen water, the dominant order was 6:2 FTS > PFCAs > PFSAs; in bathroom water, it was PFCAs ≥ 6:2 FTS > PFSAs; and in laundry water, it was PFCAs > 6:2 FTS ≥ PFSAs.

**Figure 3:**
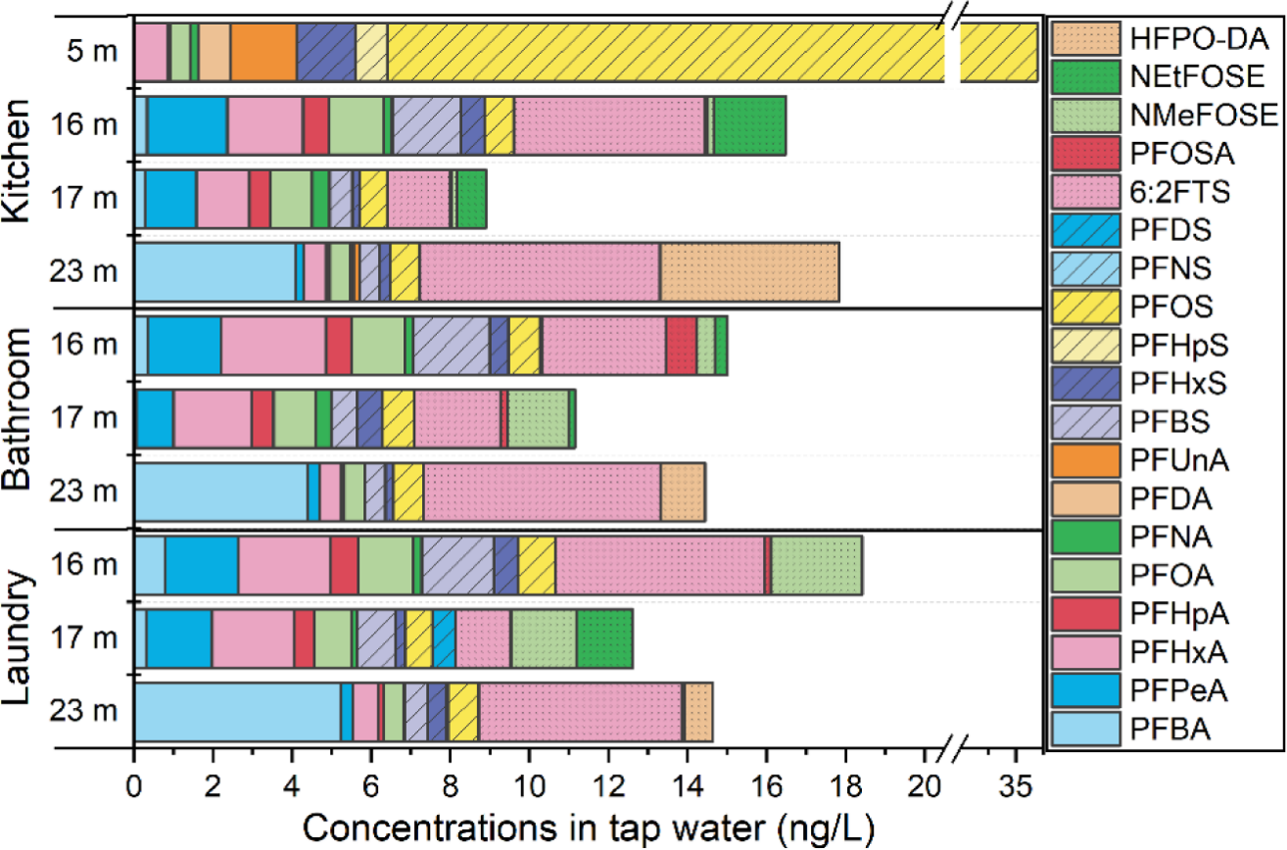
PFAS concentrations (ng/L) across different faucet locations.

A “first-draw” phenomenon was observed for PFOS and PFOA during the initial sampling event and in the data reported by MAWC (https://www.mawc.org/pfas). This refers to higher concentrations of PFAS in the first draw compared to the second draw taken after 5 min of flushing the tap, suggesting that at times closer to the incident there may have been accumulation of PFAS within the water distribution pipes. However, this phenomenon was not evident in subsequent sampling events.

### PFAS in Aerators and Filters

Total masses of detected PFAS extracted from aerators are presented in Figure 4A. Note that the aerator samples were included in sampling events 2 through 4 (16, 17, and 23 months after the incident), but not in the initial sampling event. A substantial decrease in the levels of each detected PFAS was observed over time after the incident, consistent with the trend observed in tap water samples. Both PFCAs and PFSAs were detected in all aerator extracts. Notably, HFPO–DA was detected in 5 out of 18 aerator extracts, a detection rate higher than that observed in tap water samples (<10%). No other types of PFAS were detected in the aerator samples. During sampling, we noticed that the aerators were mostly made of stainless steel, with some incorporating plastic or rubber components. One aerator was identified as having a PTFE component (gasket). However, no elevated levels of PFAAs were detected in any samples (tap water or aerator extracts) from that household. While aerators on taps can contribute to the aerosolization of water droplets, the potential for airborne dispersion of PFAS in the McKeesport area appears minimal, given the low concentrations detected in the tap water, except for the exceptionally high levels observed during initial sampling. To the best of our knowledge, this is the first study to report PFAS detection in faucet aerators. The detection of HFPO–DA in the aerator extracts raises concerns about the potential presence of emerging PFAS in these products. Further studies are needed to better understand the sources of PFAS contamination in drinking water.

**Figure 4:**
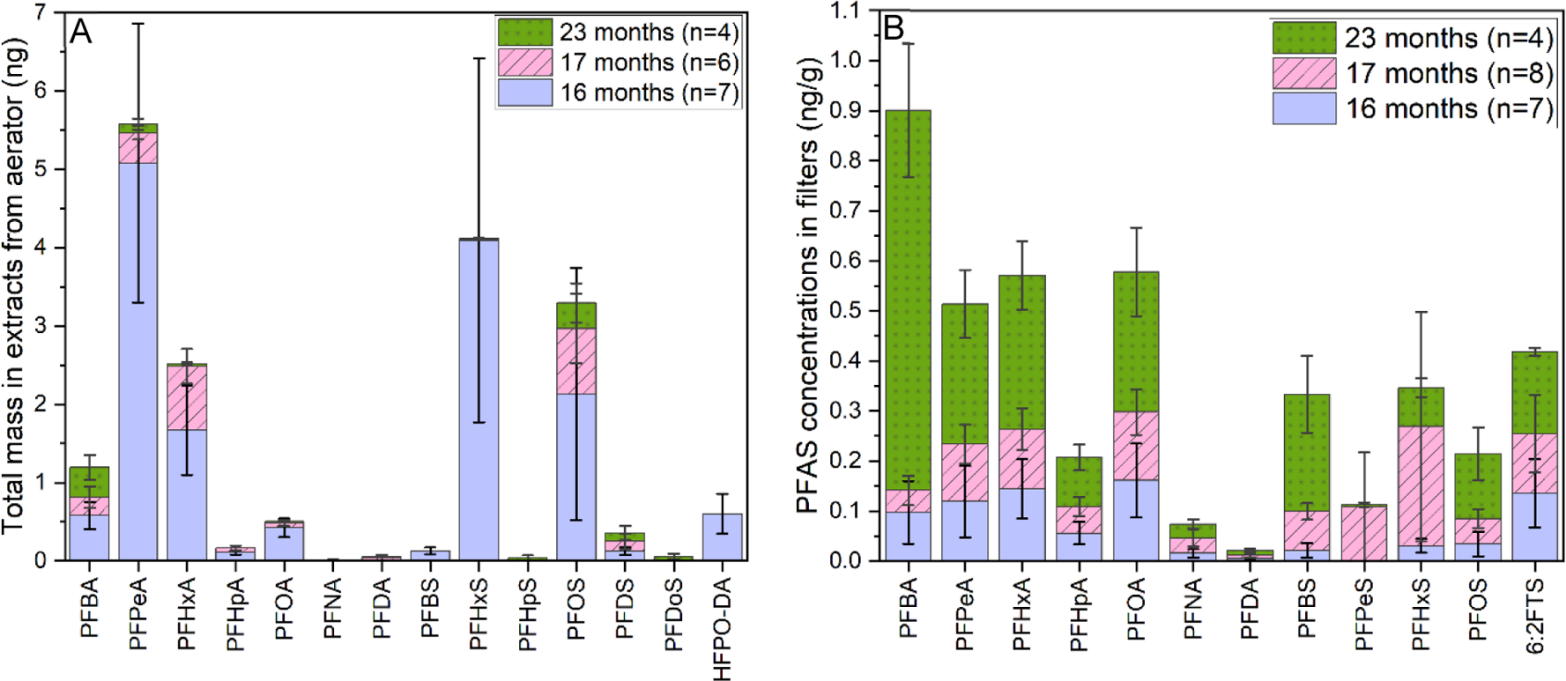
PFAS detected in (A) aerator extracts (ng) and in (B) activated carbon (ng/g) in pitcher filters (right). The number in the parentheses indicate the sample size.

The filter cartridges from the collected Zerowater pitcher filters were disassembled prior to analysis. These filters had a five-stage filtration process, which includes, from top to bottom, a mesh to remove fine particles, membrane to remove suspended solids, granular activated carbon to remove organic contaminants, an ion-exchange resin to remove metals and both organic and inorganic compounds, including charged chemicals (e.g., PFAS), and a perforated plate to remove ultrafine particles (Figure. S4). Based on the description of the materials’ filtration purpose, we analyzed PFAS in both the activated carbon and ion-exchange resin. Most PFAS levels in the ion-exchange resin were below detection limits, and therefore, the following discussion focuses solely on the PFAS found in the activated carbon. PFCAs with (C4–C10), PFSAs (C4–C6 and C8), and 6:2 FTS were detected in the activated carbon from the filters (Figure 4B). These compounds were also dominant in tap water samples in this study. It is worth noting that the concentrations of PFAS in the activated carbon are influenced by several factors, such as the PFAS concentration in the tap water, the duration of filter use, and the volume of water filtered. Due to the variability in filter usage management by participants, it is difficult to infer if the trends in household tap concentrations over time from the detected PFAS concentrations in filters. For example, elevated levels of PFAS were detected during the final sampling event (Figure 4B), and the concentrations of most PFSAs (e.g., PFBS, PFPeS, PFHxS, and PFOS) were higher in the third sampling event (17 months after the incident) compared to the second sampling event (16 months after the incident).

To evaluate the efficacy of PFAS removal by pitcher filters, we analyzed four water samples after two types of filtration were used (a pitcher filter and a tap-mounted filter from the same manufacturer as the filters analyzed above). Only trace levels of PFBA, PFBS, and 6:2 FTS were detected (Table S4), suggesting that the filter cartridges collected from the studied households could remove these PFAS. Additionally, the low detection of PFAS in the ion-exchange resin indicates that the filters still retained some capacity for PFAS removal. Previous studies have shown that granular activated carbon is more effective at removing long-chain PFAS compared to short-chain PFAS^28,29^ with sorption primarily driven by hydrophobic and electrostatic interactions. Other studies have documented that short-chain PFAS are more effectively captured by ion exchange resins.^30,31^ Therefore, dual-media filters, such as those examined in this study, could theoretically be effective in capturing a suite of PFAS. Consistent with this expectation, the majority of PFAS were effectively removed by pitcher filter filtration in this study. However, Sadia et al.^28^ reported that the removal efficiency of PFAS by granular activated carbon was limited under environmentally relevant conditions. It is important to highlight the variability in filter usage by the participants as mentioned earlier, which could impact the efficacy of pitcher filtration for PFAS removal (e.g., frequency of filter exchange). If filters were not replaced regularly, they could become a source of PFAS once saturated with absorbed contaminants.

### Beyond the Data: Community Communication

In our survey, 100% of McKeesport residents reported using municipal water, with some indicating that they filter the tap water before use. All participants expressed concerns about the water quality, not only with regard to potential PFAS contamination but also in general, following the incident. Some residents noted the presence of abnormal odors in their water after the incident, although these odors dissipated over time. Many residents shared that they felt uncertain about the safety of their water, even after the odors faded, highlighting the lasting anxiety caused by the contamination.

Our survey revealed that none of the participants reported new health issues, nor did any existing health conditions appear to worsen within 5–23 months following the incident. However, many participants conveyed an ongoing sense of unease, noting that they felt disconnected from the decision-making process and lacked clear communication from local authorities regarding the long-term health implications of PFAS exposure. This lack of transparency compounded their concerns, particularly among vulnerable populations such as the elderly and those with pre-existing health conditions.

According to US Census data, the City of McKeesport has a median age of 40 and a poverty rate of nearly 33%, and more than 50% of the populations are renters (based on data from 2019 to 2023). Renting can reduce resident control, perceived or actual, of household plumbing materials and maintenance. In this effort we partnered with community members and the Pittsburgh-based nonprofit, Women for a Healthy Environment, who together played key roles in advocating for increased attention to the community’s needs. We organized meetings to communicate with residents on sampling results and on the potential health implications of PFAS exposure. With guidance from our community liaisons, we translated our findings into clear, accessible language, and provided actionable information on exposure mitigation strategies (e.g., filter efficacy). The residents initially showed strong interest in these events, but engagement declined over time as life gradually returned to normal. In some cases, unfortunately, it was evident that residents had lost trust in their water quality, and this would not be easily regained.

## Conclusions

This work highlights the importance of consistent, transparent, and proactive communication, and underscores the need for clear guidance on cleaning and flushing in-house plumbing to support recovery from chemical release incidents, particularly in communities already facing social and environmental challenges. The findings further emphasize the importance of continued engagement and trust-building, long after the immediate crisis. Moving forward, it is essential to ensure that residents’ voices remain at the forefront of the conversation and that their concerns are continuously addressed through sustained community outreach.

The frequency of environmental incidents has risen in recent years,^32^ disproportionately affecting vulnerable populations in remote and low-income communities. These communities often face greater exposure to environmental hazards while lacking the resources to mitigate or recover from their effects. Such disparity underscores the urgent need for environmental justice frameworks that address both the unequal distribution of risks and access to necessary resources. In McKeesport, although the immediate contamination issue was resolved, exposure to high levels of PFAS could have occurred during the first few days following the incident. Given the long half-lives of several PFAS, the potential for bioaccumulation is a significant concern. Moreover, this was not the first water quality concern the community had experienced. Therefore, sustained water monitoring and proactive measures are critically important to safeguard human health.

## Supplementary Material



## Notes

Additional details of chemicals and sources, sample preparation, instrumental analysis, quality control, and data analysis can be found in the Supporting Information (PDF)
