# Supplementary material for: Per and Polyfluoroalkyl Substances in Tap Water Following an Accidental Release of Fire-Fighting Foam into the Drinking Water System in McKeesport, Pennsylvania, USA
Source: ACS ES T Water. 2026 Feb 16;6(3):1851–8. doi: 10.1021/acsestwater.5c01358 (PMC12993855; doi:10.1021/acsestwater.5c01358)
Supplement: Supplementary file 1 [file ew5c01358_si_001.pdf]

Supporting Information for:

Per- and Polyfluoroalkyl Substances in Tap Water Following an Accidental Release of Fire-fighting Foam into the Drinking Water System in McKeesport, Pennsylvania, USA

Shan Niu,[a,b] Ruiwen Chen,[b] Aaron Winchell,[b] and Carla Ng[b,c]*

[a]Advanced Interdisciplinary Institute of Environment and Ecology, Guangdong Provincial Key Laboratory of Wastewater Information Analysis and Early Warning, School of Technology for Sustainability, Beijing Normal University, Zhuhai 519087, China

[b]Department of Civil and Environmental Engineering, University of Pittsburgh, Pittsburgh, PA 15261, USA.

[c]Department of Environmental and Occupational Health, University of Pittsburgh, Pittsburgh, PA 15261, USA

*Corresponding Author:

203 Benedum Hall, 3700 O'Hara Street, Pittsburgh, PA 15261

Fax: +1-412-624-0135

Email address: carla.ng@pitt.edu

Table of Contents

Section 1. Chemicals, sample preparation, and instrumental analysis 3

Materials and Chemicals 3

Sample preparation 3

Instrumental analysis 4

Quality assurance and quality control 5

Table S1. PFAS analytes, classes, limits of detection (LOD), and amounts of spiked PFAS for the method validation. 6

Table S2. Extracted internal standards and injection internal standards used for PFAS quantification. 8

Table S3. Descriptive concentrations of target PFAS in tap water (ng/L), aerator (ng/sample), and granulated activated carbon dissembled from the filter (ng/g). 9

Table S4. Concentrations (ng/L) of PFBA, PFBS, and 6:2 FTS after two types of filtrations..... 11

Figure S1. PFAS Map published by Municipal Authority of Westmoreland County (MAWC)…12

Figure S2. Facebook post from MAWC updating residents on Do Not Use Advisory…………..13

Figure S3. PFAS concentrations published my MAWC following incident……………………..14

Figure S4. Description of the five-stage filter cartridge from Zerowater ………………….……15

Section 1. Chemicals, sample preparation, and instrumental analysis

Materials and Chemicals

High-density polyethylene (HDPE) bottles (500 mL and 1000 mL) for tap water sampling and polypropylene (PP) tubes for aerator extract sampling were purchased from Fisher Scientific (Pittsburgh, PA, USA). Sampling containers were pre-cleaned by rinsing with deionized water followed by methanol, and then air dried before use. Pitcher filters and replacement cartridges were obtained from ZeroWater (https://www.zerowater.com) and local retail outlets. Methanol (HPLC grade) and ammonium hydroxide (Optima grade) were purchased from Fisher Scientific. Native and mass-labeled per- and polyfluoroalkyl substance (PFAS) analytical standards, as listed in Tables S1 and S2, were purchased from Wellington Laboratories (ON, Canada).

Sample preparation

Tap water samples

The tap water samples were prepared following the US EPA Draft 2 Method 1633 with modifications. The sample volume was determined by weighing the full sample bottle and then the empty bottle. An aliquot of extraction internal standards (Table S2) was spiked directly into the sample (~500 mL). Subsequently, 150 µL of glacial acetic acid was added to adjust the pH to 3 – 4, and the bottle was capped and inverted to mix thoroughly. Weak anion exchange (WAX) cartridges (6cc, 150 mg) were pre-conditioned with 15 mL of methanol with 1% ammonium hydroxide (v/v), followed by 5mL of 0.3M formic acid. The prepared water sample was loaded onto the conditioned WAX cartridge at a flow rate of 5 mL/min. The sample bottle was rinsed with 5 mL of deionized water twice, followed by 4 mL of 25 mM ammonium acetate buffer (pH = 4) and dried under vacuum for 5 minutes. Elution was performed in two steps: first, with 4 mL of methanol to recover neutral PFAS (Fraction 1), and second, with 8 mL of 2% ammonium hydroxide in methanol to elute ionic PFAS (Fraction 2). The combined eluates were concentrated to 200 µL. Extracts were centrifuged, and 100 µL of the supernatant was transferred to an LC vial for targeted analysis. Injection standards were added to the 100 µL extract for targeted analysis, and the solution was mixed prior to instrumental analysis.

Aerator extracts

Approximately 10 mL of methanol was used to extract PFAS from the aerator samples. The extracts were then concentrated to 100 μL and transferred to LC vials for instrumental analysis.

Filter cartridge samples

The collected pitcher filter cartridges were disassembled to separate the activated carbon and resin. The activated carbon or resin was homogenized with a precleaned stainless steel spoon, and approximately 5g of the homogenized activated carbon or resin sample was transferred into a clean 50 mL polypropylene centrifuge tube. Prior to extraction, 11 isotopically labeled PFAS were spiked as extraction surrogates into each sample tube (see Table S2 for compound list and the amount spiked). PFAS were extracted three times with 15 mL of 0.3% NH_4OH/methanol by ultrasonication for 15 minutes. Extracts were then combined, concentrated to 250 μL under N_2, filtered through disposable syringe filters, and 100 μL of the final extract was transferred to an LC vial for targeted analysis. Injection standards consisting of 7 isotopically labeled PFAS (Table S2) were then added to the 100 μL extract for instrumental analysis.

Instrumental analysis

Quantification of PFAS followed US EPA Draft 2 Method 1633 using ultra-high performance liquid chromatography coupled with triple quadrupole mass spectrometer (UHPLC-MS/MS) equipped with an electrospray ionization source in negative-ion mode (Thermo Scientific Vanquish Flex and TSQ Quantis, Waltham, MA USA). Separation was performed on a C18 column (1.7 μm, 50 × 2.1 mm; Waters Acquity UPLC). Mobile phases A and B consisted of 20 mM ammonium acetate and methanol, respectively, at a flow rate of 0.25 mL/min. The gradient profile was: 5% B at the start, increasing to 50% over 3 minutes, 80% over 11 minutes, 95% over 14 minutes, and then returning to the initial 5% over 1 minute, held for 3 minutes. Mass spectrometry was performed in multiple reaction monitoring (MRM) mode, and parameters were adapted from Sanan and Magnuson (2020). Electrospray ionization (ESI) in negative mode was employed, with settings at 2.5 kV for spray voltage, 325 °C for the ion transfer tube temperature, and 300 °C for the vaporizer temperature.

Quality assurance and quality control

HDPE sampling bottles, new filter replacements, and PP tubes were confirmed to have no detectable PFAS before use. Method validation was conducted prior to analyzing the samples by spiking native PFAS (ranging from 2 to 20 ng depending on the analyte, Table S1) and isotopic PFAS (Table S2) as surrogates into deionized water. Recoveries of the native PFAS and the surrogates ranged from 71.4% to 108%. The limits of detection (LODs) for PFAS were established as the lowest concentrations meeting a signal-to-noise ratio of 3:1 on the instrument and ranged from 0.1 to 2.5 ng/mL (Table S1). Sample analysis was organized into batches of approximately 10 samples each. Within each batch, one laboratory blank was included. Trace amounts of PFBA and PFBS were detected in blank samples, with concentrations ranging from 0.2 to 0.4 ng/sample. The recoveries of PFAS surrogates ranged from 45 − 127%.

Table S1. PFAS analytes, classes, limits of detection (LOD), and amounts of spiked PFAS for method validation.

Class[1]	Analyte	Name	CAS number	Chain length[2]	LOD[3] (ng/L)	Amount spike (ng)
Perfluoroalkyl carboxylic acids (PFCAs)	PFBA	Perfluorobutanoic acid	375-22-4	C4	0.40	0.8
	PFPeA	Perfluoropentanoic acid	2706-90-3	C5	0.20	0.4
	PFHxA	Perfluorohexanoic acid	307-24-4	C6	0.10	0.2
	PFHpA	Perfluoroheptanoic acid	375-85-9	C7	0.10	0.2
	PFOA	Perfluorooctanoic acid	335-67-1	C8	0.10	0.2
	PFNA	Perfluorononanoic acid	375-95-1	C9	0.10	0.2
	PFDA	Perfluorodecanoic acid	335-76-2	C10	0.10	0.2
	PFUnA	Perfluoroundecanoic acid	2058-94-8	C11	0.10	0.2
	PFDoA	Perfluorododecanoic acid	307-55-1	C12	0.10	0.2
	PFTrDA	Perfluorotridecanoic acid	72629-94-8	C13	0.10	0.2
	PFTeDA	Perfluorotetradecanoic acid	376-06-7	C14	0.10	0.2
Perfluoroalkyl sulfonic acids (PFSAs)	PFBS	Perfluorobutanesulfonic acid	375-73-5	C4	0.10	0.2
	PFPeS	Perfluoropentanesulfonic acid	2706-91-4	C5	0.10	0.2
	PFHxS	Perfluorohexanesulfonic acid	355-46-4	C6	0.10	0.2
	PFHpS	Perfluoroheptanesulfonic acid	375-92-8	C7	0.10	0.2
	PFOS	Perfluorooctanesulfonic acid	1763-23-1	C8	0.10	0.2
	PFNS	Perfluorononanesulfonic acid	68259-12-1	C9	0.10	0.2
	PFDS	Perfluorodecanesulfonic acid	335-77-3	C10	0.10	0.2
	PFDoS	Perfluorododecanesulfonic acid	79780-39-5	C12	0.10	0.2
Fluorotelomer sulfonic acids	4:2FTS	4:2 Fluorotelomer sulfonic acid	757124-72-4	C6	0.40	0.8
	6:2FTS	6:2 Fluorotelomer sulfonic acid	27619-97-2	C8	0.40	0.8
	8:2FTS	8:2 Fluorotelomer sulfonic acid	39108-34-4	C10	0.40	0.8
Perfluorooctane sulfonamides	PFOSA	Perfluorooctanesulfonamide	754-91-6	C8	0.10	0.2
	NMeFOSA	N-Methylperfluorooctanesulfonamide	31506-32-8	C9	0.10	0.2
	NEtFOSA	N-Ethylperfluorooctanesulfonamide	4151-50-2	C10	0.10	0.2
Perfluorooctane sulfonamidoacetic acids	NMeFOSAA	N-Methylperfluorooctanesulfonamidoacetate	2355-31-9	C11	0.10	0.2
	NEtFOSAA	N-Ethylperfluorooctanesulfonamidoacetate	2991-50-6	C12	0.10	0.2
Perfluorooctane sulfonamide ethanols	NMeFOSE	N-Methyl perfluorooctanesulfonamidoethanesulfonate	24448-09-7	C11	1.0	2
	NEtFOSE	N-Ethyl perfluorooctanesulfonamidoethanesulfonate	1691-99-2	C12	1.0	2
Per- and polyfluoroether carboxylic acids	PFMPA	Perfluoro-3-methoxypropanoic acid	377-73-1	C4	0.20	0.8
	PFMBA	Perfluoro-4-methoxybutanoic acid	863090-89-5	C5	0.20	0.8
	NFDHA	Nonafluoro-3,6-dioxaheptanoic acid	151772-58-6	C5	0.20	0.8

	HFPO-DA	Hexafluoropropylene oxide dimer acid	13252-13-6	C6	0.40	0.8
	ADONA	4,8-Dioxa-3*H*-perfluorononanoic acid	919005-14-4	C7	0.40	0.8
Ether sulfonic acids	PFEESA	Perfluoro(2-ethoxyethane)sulfonic acid	113507-82-7	C4	0.40	0.8
	9Cl-PF3ONS	9-Chlorohexadecafluoro-3-oxononane-1-sulfonic acid	756426-58-1	C8	0.40	0.8
	11Cl-PF3OUdS	11-Chloroeicosafluoro-3-oxaundecane-1-sulfonic acid	763051-92-9	C10	0.40	0.8
Fluorotelomer carboxylic acids	3:3FTCA	3-Perfluoropropyl propanoic acid	356-02-5	C6	0.50	0.4
	5:3FTCA	2*H*,2*H*,3*H*,3*H*-Perfluorooctanoic acid	914637-49-3	C8	2.5	2
	7:3FTCA	3-Perfluoroheptyl propanoic acid	812-70-4	C10	2.5	2

1. Table ordered by PFAS class and chain length.
2. Chain length is based on the number of carbons (not necessarily fluorinated carbons) in the chemical structure.
3. LOD, limit of detection based on EPA 821-R-16-006.

Table S2. Extraction internal standards and injection internal standards used for PFAS quantification.

Full name	Abbreviation	Amount Spiked (ng)
Extraction internal standards		
Perfluoro-n-[$^{13}C_4$]butanoic acid	$^{13}C_4$-PFBA	0.8
Perfluoro-n-[$^{13}C_5$]pentanoic acid	$^{13}C_5$-PFPeA	0.4
Perfluoro-n-[1,2,3,4,6-$^{13}C_5$]hexanoic acid	$^{13}C_5$-PFHxA	0.2
Perfluoro-n-[1,2,3,4-$^{13}C_4$]heptanoic acid	$^{13}C_4$-PFHpA	0.2
Perfluoro-n-[$^{13}C_8$]octanoic acid	$^{13}C_8$-PFOA	0.2
Perfluoro-n-[$^{13}C_9$]nonanoic acid	$^{13}C_9$-PFNA	0.1
Perfluoro-n-[1,2,3,4,5,6-$^{13}C_6$]decanoic acid	$^{13}C_6$-PFDA	0.1
Perfluoro-n-[1,2,3,4,5,6,7-$^{13}C_7$]undecanoic acid	$^{13}C_7$-PFUnA	0.1
Perfluoro-n-[1,2-$^{13}C_2$]dodecanoic acid	$^{13}C_2$-PFDoA	0.1
Perfluoro-n-[1,2-$^{13}C_2$]tetradecanoic acid	$^{13}C_2$-PFTeDA	0.1
Perfluoro-1-[2,3,4-$^{13}C_3$]butanesulfonic acid	$^{13}C_3$-PFBS	0.2
Perfluoro-1-[1,2,3-$^{13}C_3$]hexanesulfonic acid	$^{13}C_3$-PFHxS	0.2
Perfluoro-1-[$^{13}C_8$]octanesulfonic acid	$^{13}C_8$-PFOS	0.2
1*H*,1*H*,2*H*,2*H*-Perfluoro-1-[1,2-$^{13}C_2$]hexane sulfonic acid	$^{13}C_2$-4:2FTS	0.4
1*H*,1*H*,2*H*,2*H*-Perfluoro-1-[1,2-$^{13}C_2$]octane sulfonic acid	$^{13}C_2$-4:2FTS	0.4
1*H*,1*H*,2*H*,2*H*-Perfluoro-1-[1,2-$^{13}C_2$]decane sulfonic acid	$^{13}C_2$-8:2FTS	0.4
Perfluoro-1-[$^{13}C_8$]octanesulfonamide	$^{13}C_8$-PFOSA	0.2
N-methyl-d_3-perfluoro-1-octanesulfonamide	D_3-NMeFOSA	0.2
N-ethyl-d_5-perfluoro-1-octanesulfonamide	D_5-NEtFOSA	0.2
N-methyl-d_3-perfluoro-1-octanesulfonamidoacetic acid	D_3-NMeFOSAA	0.4
N-ethyl-d_5-perfluoro-1-octanesulfonamidoacetic acid	D_5-NEtFOSAA	0.4
N-methyl-d_7-perfluorooctanesulfonamidoethanol	D_7-NMeFOSE	2.0
N-ethyl-d_9-perfluorooctanesulfonamidoethanol	D_9-NEtFOSE	2.0
Tetrafluoro-2-heptafluoropropoxy-13C3-propanoic acid	$^{13}C_3$-HFPO-DA	0.8
Injection internal standards		
Perfluoro-n-[2,3,4-$^{13}C_3$]butanoic acid	$^{13}C_3$-PFBA	0.4
Perfluoro-n-[1,2-$^{13}C_2$]hexanoic acid	$^{13}C_2$-PFHxA	0.2
Perfluoro-n-[1,2,3,4-$^{13}C_4$]octanoic acid	$^{13}C_4$-PFOA	0.2
Perfluoro-n-[1,2,3,4,5-$^{13}C_5$] nonanoic acid	$^{13}C_5$-PFNA	0.1
Perfluoro-n-[1,2-$^{13}C_2$]decanoic acid	$^{13}C_2$-PFDA	0.1
Perfluoro-1-hexane[$^{18}O_2$]sulfonic acid	$^{18}O_2$-PFHxS	0.2
Perfluoro-n-[1,2,3,4-$^{13}C_4$]octanesulfonic acid	$^{13}C_4$-PFOS	0.2

Table S3. Summary of measured concentrations of target PFAS in tap water (ng/L), aerator (ng/sample), and granulated activated carbon dissembled from the filter (ng/g).

Analyte	Tap water (n=150)			Aerator (n=17)			Activated carbon (n=19)		
	Detection frequency (%)	Range	50th	Detection frequency (%)	Range	50th	Detection frequency (%)	Range	50th
PFBA	32	ND – 10.3	ND	76	ND – 1.33	0.27	42	ND – 1.00	ND
PFPeA	74	ND – 9.03	1.09	94	ND – 6.50	0.51	63	ND – 0.48	0.10
PFHxA	97	ND – 8.57	1.24	88	ND – 4.87	0.67	79	ND – 0.45	0.12
PFHpA	79	ND – 2.09	0.39	59	ND – 0.22	0.06	79	ND – 0.16	0.034
PFOA	90	ND – 3.13	0.95	71	ND – 1.09	0.06	84	ND – 0.49	0.12
PFNA	74	ND – 4.18	0.16	5.9	ND – 0.05	ND	47	ND – 0.14	ND
PFDA	13	ND – 6.14	ND	18	ND – 0.22	ND	37	ND – 0.03	ND
PFUnA	6.7	ND – 3.24	ND	0	ND – ND	ND	0	ND – ND	ND
PFDoA	3.7	ND – 2.83	ND	0	ND – ND	ND	0	ND – ND	ND
PFTrDA	0.74	ND – 1.94	ND	0	ND – ND	ND	0	ND – ND	ND
PFTeDA	0.74	ND – 0.11	ND	0	ND – ND	ND	0	ND – ND	ND
PFBS	78	ND – 3.66	0.76	29	ND – 0.28	ND	74	ND – 0.42	0.05
PFPeS	0	ND – ND	ND	0	ND – ND	ND	11	ND – 0.87	ND
PFHxS	89	ND – 8.61	0.30	53	ND – 9.08	0.02	58	ND – 1.84	0.03
PFHpS	9.6	ND – 4.16	ND	5.9	ND – 0.14	ND	0	ND – ND	ND
PFOS	90	ND – 254	0.73	82	0.97 – 6.73	0.43	58	ND – 0.24	0.02
PFNS	1.5	ND – 0.26	ND	0	ND – ND	ND	0	ND – ND	ND
PFDS	8.9	ND – 7.46	ND	65	ND – 0.62	0.06	0	ND – ND	ND
PFDoS	0.74	ND – 0.17	ND	12	ND – 0.14	0.05	0	ND – ND	ND
4:2FTS	0	ND – 0.20	ND	0	ND – ND	ND	0	ND – ND	ND
6:2FTS	73	ND – 21.5	1.43	0	ND – ND	ND	53	ND – 0.50	0.03
8:2FTS	0	ND – 0.20	ND	0	ND – ND	ND	0	ND – ND	ND
PFOSA	8.3	ND – 1.1	ND	0	ND – ND	ND	0	ND – ND	ND
NMeFOSA	4.7	ND – 0.5	ND	0	ND – ND	ND	0	ND – ND	ND
NEtFOSA	4.7	ND – 0.6	ND	0	ND – ND	ND	0	ND – ND	ND
NMeFOSAA	2.5	ND – 0.72	ND	0	ND – ND	ND	0	ND – ND	ND
NEtFOSAA	0	ND – ND	ND	0	ND – ND	ND	0	ND – ND	ND
NMeFOSE	9.2	ND – 0.28	ND	0	ND – ND	ND	0	ND – ND	ND
NEtFOSE	7.5	ND – 0.19	ND	0	ND – ND	ND	0	ND – ND	ND
HFPO-DA	9.2	ND – 3.88	ND	29	ND – 1.73	ND	0	ND – ND	ND
ADONA	0.83	ND – 0.40	ND	0	ND – ND	ND	0	ND – ND	ND
PFMPA	0	ND – ND	ND	0	ND – ND	ND	0	ND – ND	ND
PFMBA	0	ND – ND	ND	0	ND – ND	ND	0	ND – ND	ND
NFDHA	0	ND – ND	ND	0	ND – ND	ND	0	ND – ND	ND

9Cl-PF3ONS	0	ND – ND	ND	0	ND – ND	ND	0	ND – ND	ND
11Cl-PF3OUdS	0	ND – ND	ND	0	ND – ND	ND	0	ND – ND	ND
PFEESA	0	ND – ND	ND	0	ND – ND	ND	0	ND – ND	ND
3:3FTCA	0	ND – ND	ND	0	ND – ND	ND	0	ND – ND	ND
5:3FTCA	0	ND – ND	ND	0	ND – ND	ND	0	ND – ND	ND
7:3FTCA	0	ND – ND	ND	0	ND – ND	ND	0	ND – ND	ND

Table ordered by PFAS class and chain length. ND = non-detect (i.e., below limit of detection values as presented in Table S1).

Table S4. Concentrations (ng/L) of PFBA, PFBS, and 6:2 FTS after two types of filtrations

**Compound**		**PFBA**	**PFBS**	**6:2FTS**
**House 1**	Faucet filter	5.49	1.64	2.78
	Pitcher filter	3.32	1.51	3.77
**House 2**	Faucet filter	6.35	1.76	2.36
	Pitcher filter	4.23	1.71	1.82

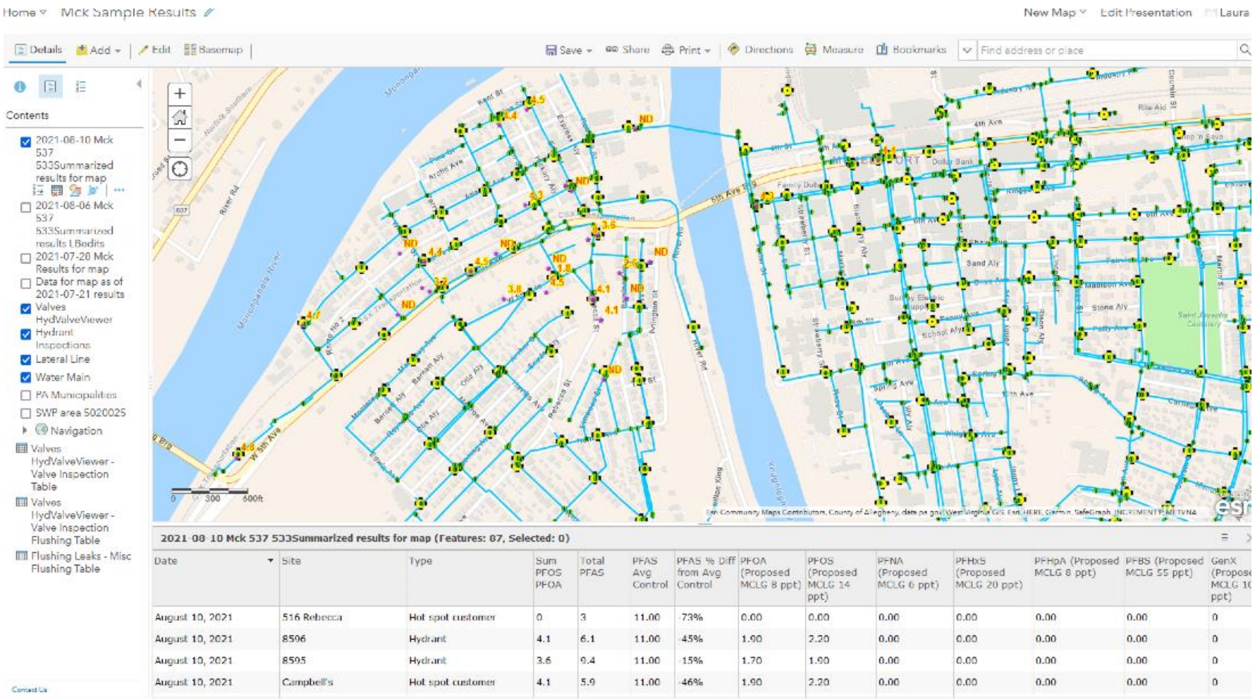

Fig S1. PFAS Map published by Municipal Authority of Westmoreland Country

(https://www.mawc.org/sites/default/files/pfas_map_8.18.21.pdf).

Municipal Authority of Westmoreland County's Post

Municipal Authority of Westmoreland County
August 12, 2021 ·

Update: MAWC Lifts Drinking Water Restrictions for Lower 10th Ward, McKeesport;
Entire Area can Use Water as Normal

McKeesport (August 12) – Following 26 days of flushing, and the 13th round of test results, MAWC lifted the final restrictions on water use for customers in Lower 10th Ward Thursday, ending restrictions that had been put in place in the early morning hours of July 17.

After 10:30 p.m. on July 16, McKeesport Fire officials informed MAWC that firefighting foam may have been introduced into the area's drinking water system during a fire on Rebecca Street.

In the early morning hours of July 17, the entire Lower 10th Ward was placed on a Do Not Use restriction. While about 500 historical accounts were once in that area, the actual number of customer accounts today is 256.

Subsequent test results of the drinking water system found constituents of PFAS-containing class B firefighting foam were present in the water system near the fire.

"This didn't need to happen. Our customers should not have been subjected to this level of inconvenience and concern, and the cost in time and money to resolve this issue was excessive," said Resident Manager Michael F. Kukura.

MAWC Isolated the area and started a cycle of flushing, sampling and testing – with employees ferrying samples to an accredited lab in Lancaster, PA to speed test results.

On July 23, following a round of customer side flushing and tests inside premises, tests confirmed contaminants. MAWC was able to isolate the area with higher results, and 34 accounts were still not able to use their water. Restrictions for the rest of the area were lifted at that time.

On July 28, with contaminant levels dropping, regulators approved an MAWC plan to further isolate the area still showing contaminants present, and fewer customers were placed on a Do Not Drink restriction.

On August 5, the affected area where customers were told not to drink their water was reduced to five addresses.

Please see our full problem corrected notice at MAWC.org

# # #

Fig S2. Social media post from MAWC updating residents on Do Not Use Advisory.

http://facebook.com/MAWCwater/posts/4386916701369067/

Date	Time	Site	Type	lat	Long	Sum PFOS PFOA	Total PFAS	PFAS Avg Control	PFOA (Proposed MCLG 8 ppt)	PFOS (Proposed MCLG 14 ppt)	PFNA (Proposed MCLG 6 ppt)	PFHxS (Proposed MCLG 20 ppt)	PFHpA (Proposed MCLG 8 ppt)	PFBS (Proposed MCLG 55 ppt)	GenX (Proposed MCLG 108 ppt)
8/10/2021	8:40	516 Rebecca	Hot spot customer	40.34972	-79.8736	0	3	11.00	0	0	0	0	0	0	0
8/10/2021	8:50	8596	Hydrant	40.34841	-79.8734	4.1	6.1	11.00	1.9	2.2	0	0	0	0	0
8/10/2021	8:35	8595	Hydrant	40.34981	-79.8733	3.6	9.4	11.00	1.7	1.9	0	0	0	0	0
8/10/2021	9:00	Campbell's	Hot spot customer	40.34795	-79.8732	4.1	5.9	11.00	1.9	2.2	0	0	0	0	0
8/9/2021	10:35	516 Rebecca	Hot spot customer	40.34972	-79.8736	1.8	9.2	11.00	1.8	0	0	0	0	0	0
8/9/2021	10:30	8596	Hydrant	40.34841	-79.8734	4.4	4.4	11.00	2.2	2.2	0	0	0	0	0
8/9/2021	10:40	8595	Hydrant	40.34981	-79.8733	4.2	12.8	11.00	2	2.2	0	0	0	0	0
8/9/2021	10:20	Campbell's	Hot spot customer	40.34795	-79.8732	5.4	14.9	11.00	2.5	2.9	0	0	0	2.1	0
8/6/2021	8:45	8596	Hydrant	40.34841	-79.8734	11.7	11.7	11.00	2.2	3	0	0	0	1.9	0
8/6/2021	8:35	8595	Hydrant	40.34981	-79.8733	16.2	16.2	11.00	2	2.6	0	0	0	1.8	0
8/6/2021	9:00	Campbells	Hot spot customer	40.34795	-79.8732	29.4	29.4	11.00	2.7	3.6	0	0	0	2	0
8/6/2021	8:40	516 Rebecca	Hot spot customer	40.34972	-79.8736	13.5	13.5	11.00	1.9	0	0	0	0	1.9	0
8/4/2021	13:45	516 Arlington	Hot spot customer	40.34922	-79.87180	**ND**	**ND**	11.00	0	0	0	0	0	0	0
8/4/2021	13:40	610 Romine	Hot spot customer	40.34899	-79.8727	2.6	2.6	11.00	0	2.6	0	0	0	0	0
8/4/2021	13:35	Romine blowoff	Now a Hydrant	40.34843	-79.8725	**ND**	**ND**	11.00	0	0	0	0	0	0	0
8/4/2021	14:15	8596	Hydrant	40.34841	-79.87345	62	62	11.00	3	4.7	0	0	0	0	0
8/4/2021	14:10	8595	Hydrant	40.34981	-79.87329	22.5	22.5	11.00	2	0	0	0	0	0	0
8/4/2021	15:00	9155	Hydrant	40.34664	-79.8731	**ND**	**ND**		0	0	0	0	0	0	0
8/3/2021	13:45	516 Arlington	Hot spot customer	40.34922	-79.87180	**ND**	**ND**		0	0	0	0	0	0	0
8/3/2021	13:40	610 Romine	Hot spot customer	40.34899	-79.8727	4.1	4.1	11.00	0	4.1	0	0	0	0	0
8/3/2021	13:35	Romine blowoff	Now a Hydrant	40.34843	-79.8725	**ND**	**ND**	11.00	0	0	0	0	0	0	0
8/3/2021	14:15	8596	Hydrant	40.34841	-79.87345	59.8	59.8	11.00	3.1	5.2	0	0	0	0	0
8/3/2021	14:10	8595	Hydrant	40.34981	-79.87329	16.6	16.6	11.00	1.8	0	0	0	0	0	0
8/3/2021	15:00	9155	Hydrant	40.34664	-79.8731	**ND**	**ND**		0	0	0	0	0	0	0
7/30/2021	7:25	516 Arlington	Hot spot customer	40.34922	-79.87180	10.7	10.7	11.00	1.8	0	0	0	0	0	0
7/30/2021	7:20	Romine blowoff	Blowoff	40.34843	-79.87248	95.6	95.6	11.00	4.2	19	2.1	0	0	0	0
7/30/2021	7:23	8596	Hydrant	40.34841	-79.87345	126.8	126.8	11.00	5.3	26	2.5	0	0	0	0
7/30/2021	7:10	8595	Hydrant	40.34981	-79.87329	2.4	2.4	11.00	0	0	0	0	0	0	0
7/30/2021	6:55	8565	Hydrant	40.35214	-79.8722	**ND**	**ND**		0	0	0	0	0	0	0
7/29/2021	7:45	516 Arlington	Hot spot customer	40.34922	-79.87180	5.7	19.1	11.00	2	3.7	0	0	0	0	0
7/29/2021	7:40	Romine blowoff	Blow off	40.34843	-79.87248	13	78.3	11.00	4.1	8.9	3.5	0	0	0	0
7/29/2021	7:50	8596	Hydrant	40.34841	-79.87345	4.3	4.3	11.00	0	4.3	0	0	0	0	0
7/29/2021	7:30	8595	Hydrant	40.34981	-79.87329	4.4	4.4	11.00	0	4.4	0	0	0	0	0
7/29/2021	8:00	8565	Hydrant	40.35214	-79.87222	**ND**	**ND**		0	0	0	0	0	0	0
7/28/2021	12:45	Romine blowoff	Blowoff	40.34843	-79.87248	8.3	18.4	11.00	2	6.3	0	0	0	0	0
7/28/2021	12:40	516 Arlington	Hot spot customer	40.34922	-79.87180	5.4	15.1	11.00	0	5.4	0	0	0	0	0
7/28/2021	13:05	8565	Background	40.35214	-79.8722	**ND**	**ND**		0	0	0	0	0	0	0
7/27/2021	0458	Romine blowoff	Blowoff	40.34843	-79.87248	9.8	57.5	11.00	2.5	7.3	0	0	0	0	0
7/27/2021	0530	8595	Hydrant	40.34981	-79.87329	5.8	18.2	11.00	0	5.8	0	0	0	0	0
7/27/2021	0555	8597	Hydrant	40.34886	-79.87459	1.8	4.5	11.00	0	1.8	0	0	0	0	0
7/27/2021	05:40	516 Arlington Ave	Hot spot customer	40.34922	-79.87180	8.8	**53**	11.0	2.2	6.6	0	0	0	0	0
7/27/2021	05:40	300 W. 6th Ave	Hot spot customer	40.34908	-79.87472	**ND**	**ND**		0	0	0	0	0	0	0
7/27/2021	06:00	8596	Hydrant	40.34841	-79.87345	10.9	**37.2**	11.0	1.9	9	0	0	0	0	0
7/24/2021	4:12	516 Arrington-first	Hot spot customer	40.34922	-79.8718	18.7	67.8	11.00	3.7	15	0	0	0	0	0
7/24/2021	4:25	516 Arlington-10 min	Hot spot customer	40.34922	-79.8718	18.6	65.7	11.00	3.6	15	0	0	0	0	0
7/24/2021	4:35	516 Arlington-20 min	Hot spot customer	40.34922	-79.8718	18.5	**62.6**	11.0	3.5	15	0	0	0	0	0
7/24/2021	4:45	Romine blowoff	Blowoff	40.34843	-79.87248	19.3	**74**	11.0	4.3	15	0	0	0	0	0
7/24/2021	5:00	8596	Hydrant	40.34841	-79.8734	5.3	**9.1**	11.0	1.8	3.5	0	0	0	0	0
7/24/2021	5:00	Campbells- first draw	Hot spot customer	40.34795	-79.8732	7.9	20.7	11.00	2.6	5.3	0	0	0	0	0
7/24/2021	5:10	Campbells- 10min	Hot spot customer	40.34795	-79.8732	5.4	**9.3**	11.0	1.9	3.5	0	0	0	0	0
7/24/2021	5:20	Campbells- 20min	Hot spot customer	40.34795	-79.8732	5.9	**5.9**	11.0	2.1	3.8	0	0	0	0	0
7/24/2021	5:15	8595	Hydrant	40.34981	-79.8733	23.6	39.6	11.00	2.6	21	0	0	0	0	0
7/24/2021	5:35	8597	Hydrant	40.34886	-79.8746	8.9	17.1	11.00	2.4	6.5	0	0	0	0	0
7/24/2021	5:30	300 west 6th-first	Hot spot customer	40.34908	-79.8747	6.7	18.8	11.00	2.6	4.1	0	0	0	0	0
7/24/2021	5:40	300 west 6th-10 min	Hot spot customer	40.34908	-79.8747	4.2	5.9	11.00	1.7	2.5	0	0	0	0	0
7/24/2021	5:50	300 west 6th-20 min	Hot spot customer	40.34908	-79.8747	4.3	4.3	11.00	2.2	2.1	0	0	0	0	0
7/21/2021	10:05	524 W 5th Ave	Customer	40.34807	-79.879	ND	ND		0	0	0	0	0	0	0
7/21/2021	10:25	506W 5th Ave	Customer	40.34866	-79.8784	ND	ND		0	0	0	0	0	0	0
7/21/2021	11:10	8596 US	US hydrant tap	40.34838	-79.8734	39.7	199.88	10.99	8.7	31	0	2.4	0	0	0
7/21/2021	11:20	8596 DS first draw	DS hydrant tap	40.34843	-79.8735	144	650	10.99	34	110	7.6	13	6.7	4.3	0
7/21/2021	11:40	506 Gross St	Customer	40.3494	-79.879	ND	**ND**		0	0	0	0	0	0	0
7/21/2021	11:35	8596 DS five min	DS hydrant tap	40.34843	-79.8735	53	283.11	10.99	12	41	2.9	2.7	0	0	0
7/21/2021	11:55	Campbells-627 Rebecca St first	Hot spot customer	40.34795	-79.8732	43.1	200.8	10.99	9.1	34	2.2	2.6	1.8	1.8	0
7/21/2021	12:00	Campbells-627 Rebecca St. five min	Hot spot customer	40.34795	-79.8732	40.8	195.66	10.99	8.8	32	1.8	2.5	1.8	1.7	0
7/21/2021	12:10	216 Pacific	Customer	40.35089	-79.8742	ND	ND		2	0	0	0	0	0	0
7/21/2021	12:30	217 Pacific	Customer	40.35078	-79.8741	ND	ND		0	0	0	0	0	0	0
7/21/2021	13:05	300 w. 6th first	Hot spot customer	40.34908	-79.8747	111	416.4	10.99	4.4	9.3	0	0	0	0	0
7/21/2021	13:10	300 w 6th 5 min	Hot spot customer	40.34908	-79.8747	7.1	24.07	10.99	3.1	4	0	0	0	0	0
7/21/2021	13:10	410 W. 5th	Customer	40.34941	-79.8762	ND	ND		0	0	0	0	0	0	0
7/21/2021	13:25	309 w 6th first	Background Port Vue	40.34856	-79.8748	2.9	11.3	10.99	1.7	0	0	0	0	1.7	0
7/21/2021	13:30	309 w 6th 5 min	Background Port Vue	40.34856	-79.8748	4.5	10.2	10.99	2.7	1.8	0	0	0	1.7	0
7/21/2021	13:40	502 W 5th ave	Customer	40.34857	-79.8781	3.2	5.8		2	0	0	0	0	0	0
7/21/2021	13:45	408 W 6th first	Background Port Vue	40.34841	-79.876	3.6	11.52	10.99	2.2	0	0	0	0	0	0
7/21/2021	13:50	408 W. 6th five min	Background Port Vue	40.34841	-79.876	3.8	10.95	10.99	2	1.8	0	0	0	0	0
7/19/2021	11:20	8005-Control	Hydrant	40.35145	-79.8653	4.4	9.3	7.27	2.4	2	0	0	0	0	0
7/19/2021	11:15	8565	Hydrant	40.35214	-79.8722	4.3	9.1	7.27	2.4	1.9	0	0	0	0	0
7/19/2021	11:40	8568	Hydrant	40.35256	-79.8753	4.5	9.4	7.27	2.4	2.1	0	0	0	0	0
7/19/2021	11:35	8056-Control	Hydrant	40.35045	-79.8687	4.3	2.1	7.27	2.4	1.9	0	0	0	0	0
7/19/2021	12:00	Alpine tank-Control	Hydrant	40.34153	-79.8556	4.9	10.4	7.27	2.6	2.3	0	0	0	0	0
7/19/2021	12:05	8570	Hydrant	40.35222	-79.8761	4.4	8.9	7.27	2.4	2	0	0	0	0	0
7/19/2021	12:33	8582	Hydrant	40.35047	-79.8754	4.3	8.7	7.27	2.3	2	0	0	0	0	0
7/19/2021	12:56	8577	Hydrant	40.34784	-79.8818	4.7	10	7.27	2.5	2.2	0	0	0	0	0
7/19/2021	13:15	8579	Hydrant	40.34922	-79.8783	4.4	8.9	7.27	2.3	2.1	0	0	0	0	0
7/19/2021	13:15	EP101	Hydrant	40.33922	-79.8602	4.4	6.2	7.27	2.2	1.8	0	0	0	0	0
7/19/2021	13:40	8594	Hydrant	40.34493	-79.8837	4.8	11.2	7.27	2.6	2.2	0	0	0	1.7	0
7/19/2021	13:28	8590.1	Hydrant	40.34901	-79.8769	4.5	9.2	7.27	2.4	2.1	0	0	0	0	0
7/19/2021	13:55	8596	Hydrant	40.34841	-79.8734	77.6	304.6	7.27	9.6	68	5.8	3.1	3.3	2.1	0

Fig S3. PFAS concentrations published my MAWC following incident https://www.mawc.org/sites/default/files/2021-08-10_mck_537_533summarized_results_1.pdf

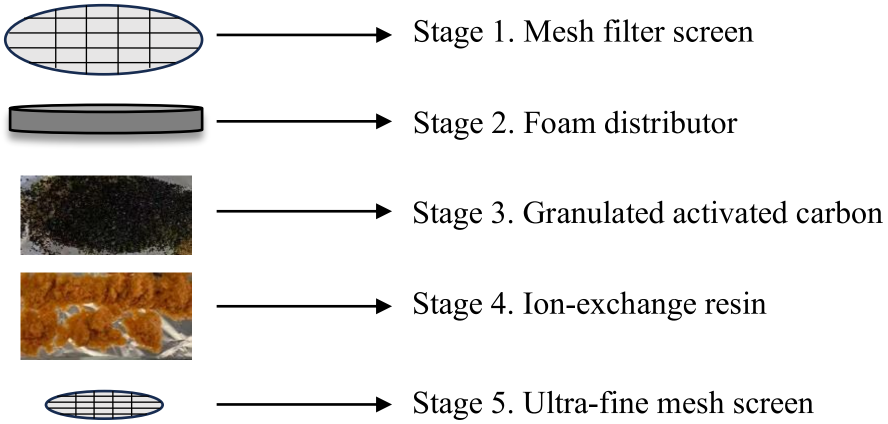

Fig S4. Description of the five-stage filter cartridge from Zerowater (www.zerowater.com)
